# The inhibitory activity of gallic acid against DNA methylation: application of gallic acid on epigenetic therapy of human cancers

**DOI:** 10.18632/oncotarget.23015

**Published:** 2017-12-07

**Authors:** Yui-Ping Weng, Pin-Feng Hung, Wen-Yen Ku, Chang-Yuan Chang, Bo-Han Wu, Ming-Han Wu, Jau-Ying Yao, Ji-Rui Yang, Chia-Huei Lee

**Affiliations:** ^1^ Graduate Institute of Biomedical Science, Chung Hwa University of Medical Technology, Tainan, Taiwan; ^2^ National Institute of Cancer Research, National Health Research Institutes, Zhunan, Taiwan

**Keywords:** gallic acid, DNA methyltransferase, chemoprevention, epigenetics, post-fermentation oolong tea extract

## Abstract

Epigenome aberrations have been observed in tobacco-associated human malignancies. (−)-epigallocatechin-3-gallate (EGCG) has been proven to modulate gene expression by targeting DNA methyltransferases (DNMTs) through a proposed mechanism involving the gallate moiety of EGCG. We show that gallic acid (GA) changes the methylome of lung cancer and pre-malignant oral cell lines and markedly reduces both nuclear and cytoplasmic DNMT1 and DNMT3B within 1 week. GA exhibits stronger cytotoxicity against the lung cancer cell line H1299 than EGCG. We found that GA reactivates the growth arrest and DNA damage-inducible 45 (GADD45) signaling pathway may through the demethylation of CCNE2 and CCNB1 in H1299 cells. To improve the epigenetic anti-cancer activities of oolong tea, we identified a fungus, *Aspergillus sojae* which can efficiently increase the GA content in oolong tea via a 2-week fermentation process. The fungus dramatically increased GA up to 44.8 fold in the post-fermentation oolong tea extract (PFOTE), resulting in enhanced demethylation effects and a significant reduction in the nuclear abundances of DNMT1, DNMT3A, and DNMT3B in lung cancer cell lines. PFOTE also showed stronger anti-proliferation activities than oolong tea extract (OTE) and increased sensitivity to cisplatin in H1299 cells. In summary, we demonstrate the potent inhibitory effects of GA on the activities of DNMTs and provide a strong scientific foundation for the use of specialized fermented oolong tea high in GA as an effective dietary intervention strategy for tobacco-associated cancers.

## INTRODUCTION

Epigenetic aberrations have been recognized as vital mechanisms leading to human cancers. Recent research has shown that cigarette smoking [1] make individuals susceptible to malignancies involving altered epigenome. DNA methylation has been recognized as a key player in the epigenetic silencing of tumor suppressor genes involved in carcinogenesis. DNA methylation patterns in mammals are mainly established by a complex interplay of three independently encoded DNA methyltransferases (DNMTs): DNMT1, DNMT3A, and DNMT3B. Aberrant DNMT expression and activity have been observed in many human malignancies, including lung and oral cancers [2, 3]. Epigenetic changes are reversible, which render them an attractive therapeutic target for human cancers. Some dietary factors may have beneficial effects on cancer susceptibility by alleviating altered epigenetic modifications. The therapeutic properties of green tea have been particularly noticed in various types of human malignancies [4]. (−)-Epigallocatechin-3-gallate (EGCG) has been recognized as the most effective anti-cancer polyphenol in green tea [4, 5]. Fang’s study [6] has demonstrated that EGCG is able to inhibit DNA methyltransferases (DNMTs) and re-activate methylation-silenced genes in human cancer cells of the colon, esophagus, and prostate. The interaction between EGCG and DNMTs has been elucidated by a molecular structural model that revealed that EGCG is well accommodated in a catalytic active pocket of DNMT1 by forming a hydrogen-bond network and, in turn, that EGCG inhibited DNMT activity in a dose-dependent and competitive inhibitory fashion. The gallate moiety of EGCG and five amino acids of DNMT1 — including Pro^1223^, Glu^1265^, Cys^1225^, Ser^1229^, and Arg^1309^ — are involved in the formation of hydrogen bonds. By such interactions, the galloyl moiety causes EGCG to have inhibitory effects on DNMT1 function by blocking the entry of the key cytosine into its active site and, in turn, preventing methylation. Furthermore, EGCG analogs without the gallic acid (GA, also known as 3,4,5-trihydroxybenzoic acid) moiety are poor inhibitors of DNMTs. These molecular structural model-based findings suggest the possibility that GA protects the epigenome from environmental stimuli by effectively inhibiting DNMT’s activities.

In the present study we aimed to assess the potential inhibitory effect of GA on the activities of DNMTs in tobacco-associated human cancers. Additionally, if GA efficiently inhibits DNMTs, we aimed to examine whether the suppressing effect of tea on altered epigenome can be improved by enrichment of GA and, in turn, provide a beverage with chemoprevention activity against tobacco-associated cancers.

## RESULTS

### GA changes genomic 5-methyl-2-deoxycytidine (5mC) content and reduces the cytoplasmic and nuclear abundance of DNMT1 and DNMT3B

**Figure 1:**
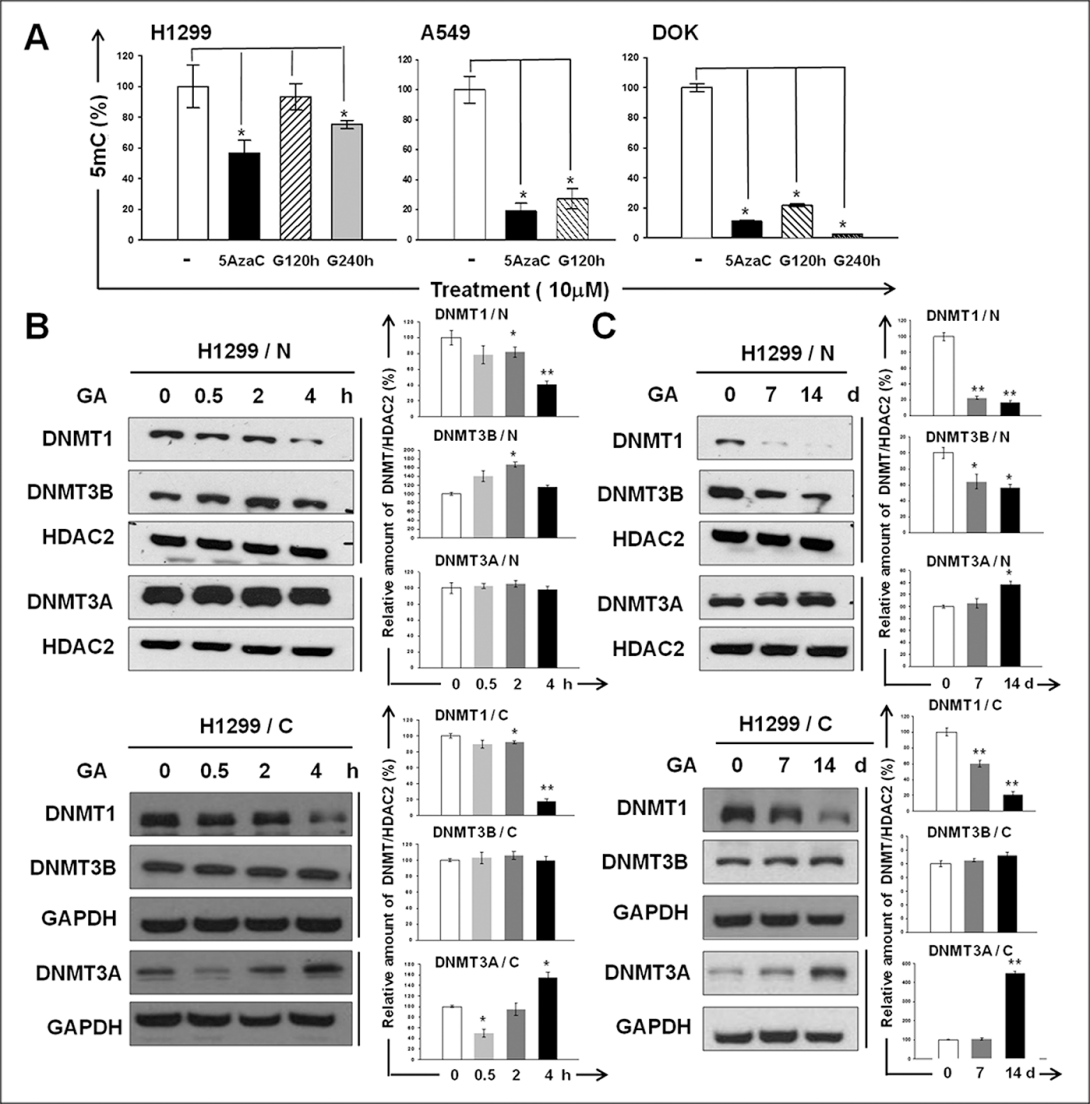
The inhibitory effect of GA on DNMTs (**A**) Genomic 5 mC content of H1299, A549, and DOK cell lines with or without GA (10 μM) treatment for 120 or 240 h. Cells treated with 5azaC (10 μM) for 120 h were used as positive controls. Data are presented as means ± SD (*n* = 3). (**B**–**C**) The abundances of the nuclear (N) and cytoplasmic (C) DNMT1, DNMT3A, and DNMT3B in GA-treated H1299 were determined using western blotting. The amount of histone deacetylase 2 (HDAC2) or GAPDH detected on the same membrane (indicated by vertical lines) was used as a loading control for normalization. The intensity for each protein band was quantified using ImageJ. The relative fold-changes after normalization are presented. Normalized quantities of DNMTs at time zero were set as 100%.

We measured 5mC in H1299, A549, and DOK after GA treatment. Compared with the untreated control, treatment of GA (10 μM) for 120 and 240 h significantly changed the genomic 5mC content in all cell lines examined (Figure 1A). To determine whether the activities of DNMTs change in response to GA treatment, we analyzed the nuclear and cytoplasmic abundance of DNMT1, DNMT3A, and DNMT3B in GA-treated H1299 cells. We observed a significant decline in both the nuclear and cytoplasmic abundance of DNMT1 after either short-term (Figure 1B) or long-term (Figure 1C) treatment of GA. Nuclear DNMT1 were barely undetectable at 5 and 7 days post GA exposure. Cytoplasmic DNMT3B remained almost unchanged after either short-term (Figure 1B) or long-term (Figure 1C) treatment of GA, whereas nuclear DNMT3B slightly increased and significantly decreased by short-term and long-term GA treatment, respectively (Figure 1B, 1C). A 4 h period of GA exposure had no effect on the nuclear DNMT3A abundance (Figure 1B) and all the other experimental conditions lead to a significant increase in cytoplasmic and nuclear DNMT3A (Figure 1B, 1C). Altogether, these results suggest that GA has profound effect on both the cytoplasmic and nuclear abundance of DNMTs, especially for DNMT1, the major DNMT expressing in H1299.

### Inhibitory effect of GA on proliferation and clonogenicity of human lung and oral cancer cell lines

**Figure 2:**
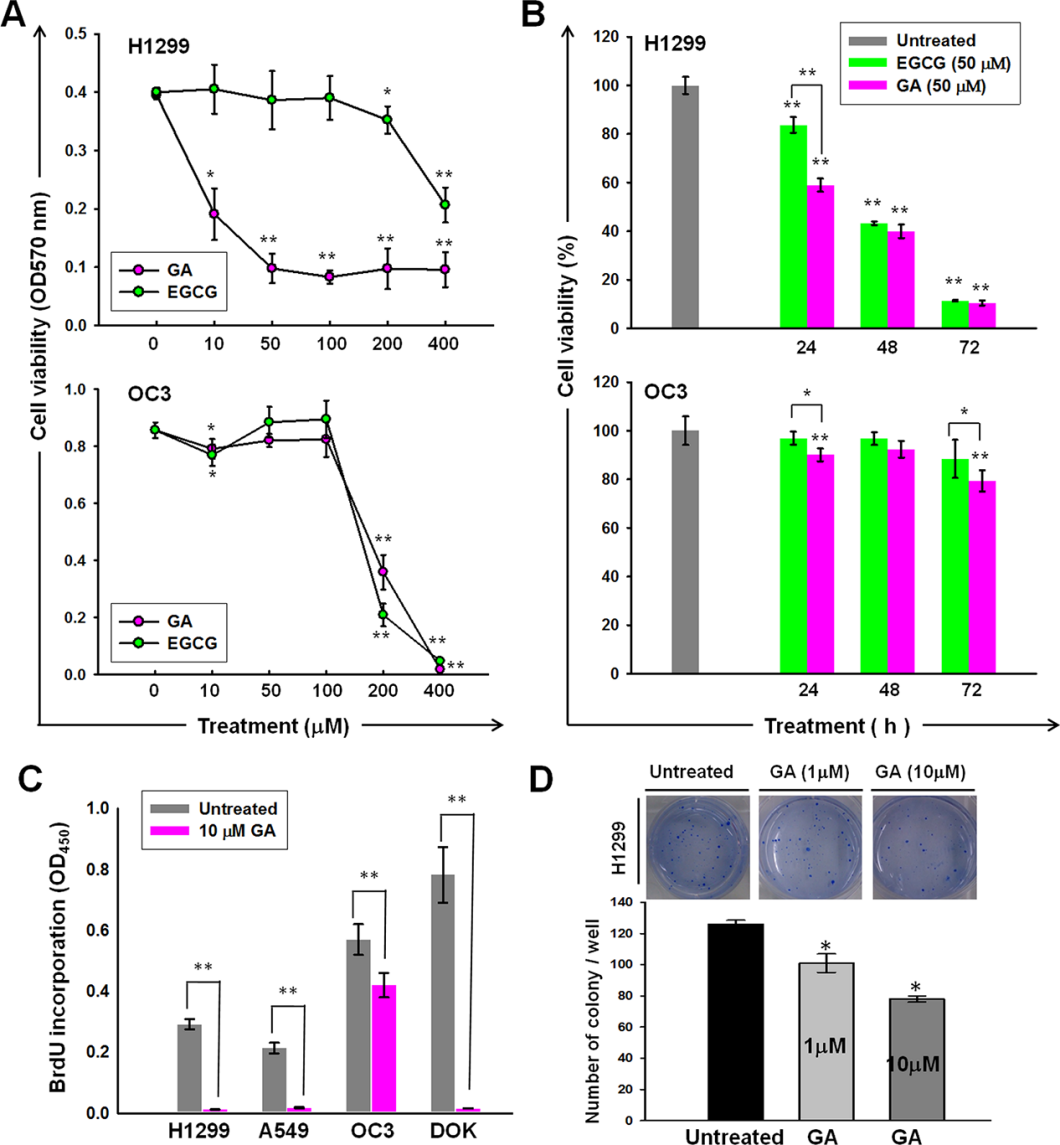
Anti-cancer activities of GA on lung and oral cancer cell lines Dose- (**A**) and time- (**B**) dependent effects of GA or EGCG on the cell viability of H1299 and OC3 cell lines. The cells were treated with GA or EGCG at the indicated concentrations for 24 h (A) or 50 mM of GA or EGCG for indicated time periods (B), followed by MTT analysis. (**C**) BrdU incorporation of A549, H1299, OC3, and DOK was inhibited by GA treatment. The cells were cultured in normal growth medium with or without GA (10 μM) treatment for 48 h, followed by BrdU assays. (**D**) GA inhibited the anchorage-independent growth of H1299. Data are presented as means ± SD (*n* = 3).

Treatment with GA or EGCG resulted in the inhibition of H1299 cell proliferation in both dose-dependent (Figure 2A) and time-dependent (Figure 2B) manners. Moreover, the cytotoxicity of GA to the H1299 cell line was significantly greater (*P* = 5.15981E-07) than that of EGCG (Figure 2A). The inhibitory effects of GA on the proliferative capacities were confirmed by BrdU incorporation (Figure 2C). As shown in Figure 2D, GA treatment inhibited the clonogenicity of H1299 cells in a dose-dependent manner.

### Identification of an anti-tumour pathway epigenetically reactivated by DNA demethylation in GA-treated lung cancer cell

**Figure 3:**
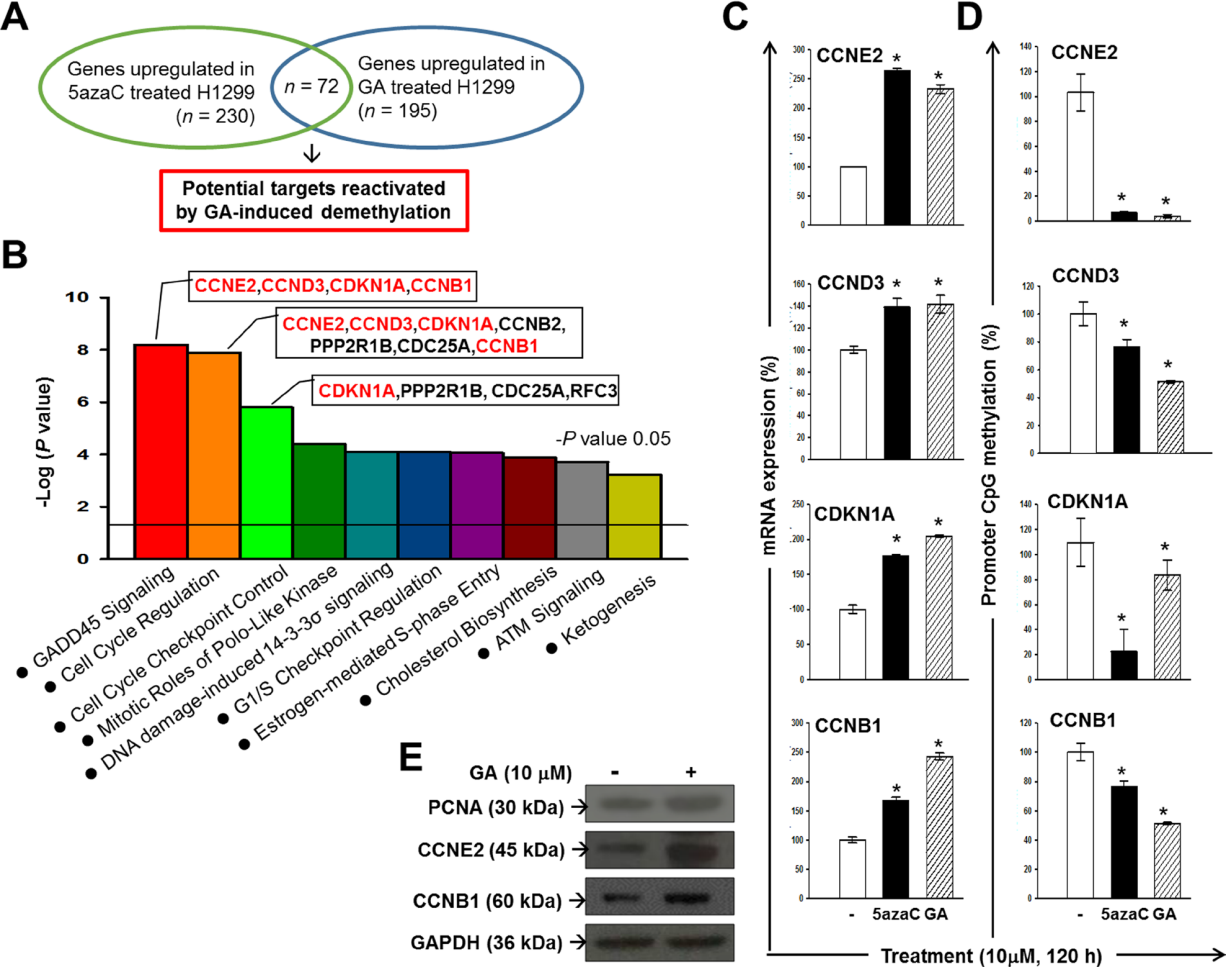
Identification of potential anti-cancer pathways reactivated by GA-induced demethylation in H1299 (**A**) An experimental design using an expression microarray platform to identify potential epigenetic targets of GA in H1299. (**B**) A graph showing the top 10 canonical pathways significantly (*P* < 0.05) reactivated in 5AzaC- and GA-treated H1299 cells. Red-colored genes are up-regulated genes mapped to the GADD45 signaling pathway in 5azaC- and GA-treated H1299 cells. Results of microarray were validated by qPCR (**C**) and qMSP analysis (**D**). (**E**) Protein expressions of PCNA, CCNE2, and CCNB1 in GA-treated H1299 cells. GAPDH was used as a loading control.

To identify the potential epigenetic targets of GA that are implicated in the anti-cancer activity of GA, we conducted an integrated analyses of gene expression profiles obtained from 5azaC- and GA-treated H1299 cells (Figure 3A). At 120 h post-treatment, a total of 383 and 546 genes were either up- or down-regulated greater than two-fold in H1299 cells grown in media containing 10 μM GA or 5AzaC, respectively. Of these, we identified 72 genes that were up-regulated by both 5AzaC and GA treatments using a Venn diagram (Figure 3A and Table 1), suggesting that these genes may increase expression by promoter demethylation during the GA treatment period. A heat map showing the relative expression of the 72 genes in response to 5AzaC and GA treatments is presented in Supplementary Figure 1. Ingenuity Pathway Analysis (IPA) classified these 72 genes into seven groups based on molecular function (Supplementary Figure 2). IPA identified 32 genes eligible for canonical pathway analysis. The top three statistically significant canonical pathways, including Growth Arrest and DNA Damage-inducible protein 45 (GADD45) signaling, cyclins and cell cycle regulation, and checkpoint proteins in cell cycle checkpoint control (Figure 3B), were all implicated in tumorigenesis. From the 72-gene list, four genes, including CCNE2, CCND3, CDKN1A, and CCNB1, accounted for 21% (4/19) of the genes involved in GADD45 signaling, which was the most significant pathway up-regulated in the GA-treated H1299 cells. These four genes also mapped to pathways related to cell cycle regulation and cell cycle checkpoint control (Figure 3B). The mRNA expressions of CCNE2, CCND3, CDKN1A, and CCNB1 were validated by qPCR (Figure 3C). Results from qMSP suggested that the reactivated expressions of CCNE2, CCND3, CDKN1A, and CCNB1 by GA may through promoter demethylation (Figure 3D). The protein expressions of CCNE2, and CCNB1 were apparently increased by GA treatment for 120 h (Figure 3E). These data suggested that the biological functions of CCNE2 and CCNB1 may contribute to the anti-cancer effect of GA in H1299 cell line.

**Table 1:** Common genes those were significantly up-regulated in response to GA and 5AzaC treatment in H1299 cells, with their respective fold-change (FC) values and *P*-values (*P*) from statistical comparison

Gene Symbol	GA (120 h)		5AzaC (120 h)	
	Fold Change	*P*-value	Fold Change	*P*-value
HSPA1B	5.25	0.00	4.89	0.03
HSPA1A	4.44	0.02	4.46	0.01
FBXL5	3.66	0.01	3.76	0.01
FAM111B	3.05	0.01	3.24	0.00
ACAT2	2.67	0.00	3.19	0.00
HSPE1	2.97	0.00	3.04	0.00
CSRP2	2.44	0.03	2.93	0.02
CCNE2	2.55	0.03	2.92	0.01
FGFR1OP	2.25	0.00	2.86	0.02
UBE2T	3.60	0.02	2.84	0.01
CDCA2	2.26	0.03	2.78	0.00
CA2	2.82	0.03	2.78	0.01
BUB1	2.28	0.01	2.70	0.01
EMP2	2.15	0.01	2.70	0.04
TGFBI	2.61	0.05	2.66	0.02
PIN4	2.10	0.00	2.66	0.02
POP1	2.39	0.01	2.64	0.05
SGK3	2.16	0.00	2.55	0.02
GLA	2.55	0.02	2.47	0.02
CHORDC1	2.02	0.02	2.44	0.00
HSPH1	2.31	0.01	2.44	0.01
PGM3	2.29	0.01	2.43	0.00
AP1S3	2.14	0.04	2.42	0.01
ERCC6L	2.47	0.00	2.41	0.00
TNFRSF10D	3.06	0.01	2.40	0.02
CDKN1A	3.52	0.03	2.39	0.04
ANLN	2.16	0.04	2.39	0.01
PPP4R4	2.45	0.02	2.37	0.01
ALYREF	2.47	0.00	2.35	0.03
FABP5	2.32	0.03	2.35	0.05
ID2	2.66	0.04	2.35	0.00
SAMHD1	2.34	0.04	2.34	0.01
TPTE	2.04	0.01	2.33	0.00
DHCR24	2.98	0.00	2.32	0.00
TTF2	2.19	0.05	2.32	0.01
KATNAL1	2.27	0.02	2.28	0.04
LYAR	2.23	0.00	2.28	0.01
CCNB2	2.26	0.02	2.27	0.02
POLE2	2.10	0.02	2.26	0.04
KIF20B	2.08	0.03	2.26	0.01
PPP2R1B	2.10	0.02	2.25	0.02
HMGCS1	2.20	0.00	2.25	0.01
DLGAP5	2.53	0.00	2.25	0.00
PSMA5	2.26	0.03	2.24	0.01
SPC25	2.33	0.03	2.22	0.01
LRRC6	2.18	0.02	2.20	0.05
POLH	2.26	0.01	2.18	0.00
KNSTRN	2.45	0.00	2.17	0.00
RFC3	2.04	0.00	2.17	0.00
KIF20A	2.48	0.01	2.16	0.01
FAM98A	2.20	0.00	2.15	0.00
TUBGCP4	2.36	0.03	2.15	0.00
SEMA6A	2.37	0.00	2.15	0.04
LMO7	2.33	0.02	2.15	0.02
INSIG1	2.59	0.01	2.15	0.03
FAM122B	2.66	0.04	2.11	0.02
IFNAR2	2.11	0.04	2.09	0.01
FHL2	2.35	0.00	2.09	0.01
HSPB11	2.18	0.03	2.09	0.01
PCNA	2.51	0.02	2.09	0.00
FAM102B	2.77	0.02	2.09	0.00
SOGA1	2.16	0.00	2.07	0.00
CDC25A	3.00	0.01	2.07	0.01
LMNB1	2.03	0.01	2.06	0.01
GLDC	2.73	0.00	2.04	0.03
CCNB1	2.08	0.04	2.04	0.00
HJURP	2.08	0.01	2.03	0.01
TRMU	2.11	0.02	2.03	0.02
TPM1	2.11	0.03	2.03	0.01
CCND3	2.34	0.04	2.02	0.02
CD83	2.58	0.03	2.00	0.03
TSPAN13	2.15	0.02	2.00	0.02

### Preparation of post-fermentation oolong tea extract (PFOTE) high in GA

**Figure 4:**
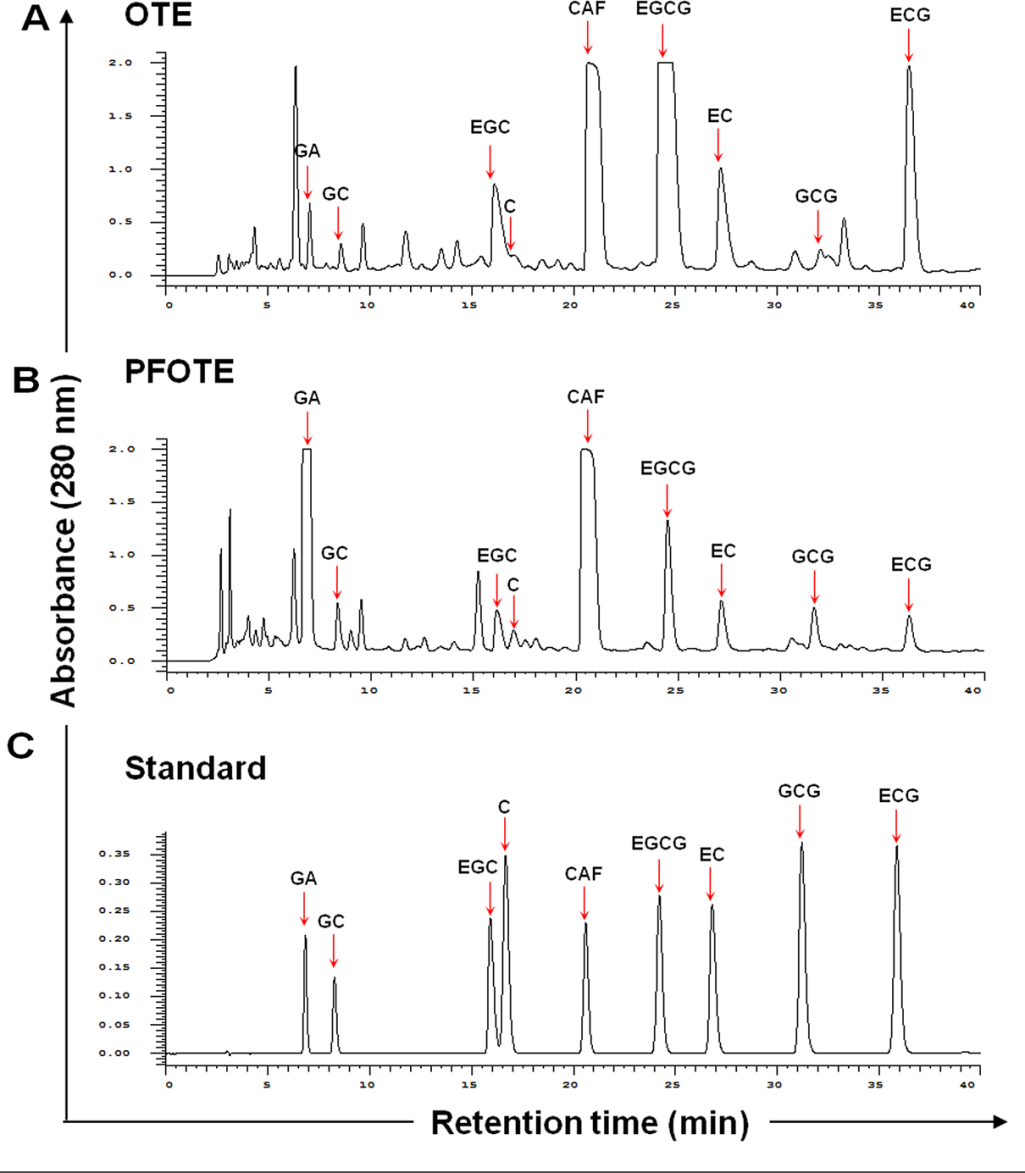
Comparison of polyphenol profile of oolong tea extract (OTE) before and after *A*. *sojae*-mediated fermentation Chromatographic patterns from HPLC analysis of OTE (**A**), PFOTE (**B**), and standard compounds [peak 1, GA; 2, GC; 3, EGC; 4, C; 5, CAF; 6, EGCG; 7, (−)-epicatechin; 8, GCG; 9, ECG] (**C**). Absorbance was monitored at 280 nm.

Given the epigenetic and anti-cancer effects of GA, we sought to enhance the chemoprevention efficacy of oolong tea by increasing its GA content. We found that *Aspergillus sojae* (*A. sojae*) was capable of generating substantial quantities of GA by a two-week rapid fermentation process. Comparison of HPLC profiles revealed that OTE contains seven catechins (GC, EGC, C, EGCG, EC, GCG, and ECG), GA, and CAF (Figure 4A). After *A. sojae*-mediated biotransformation, the GA content dramatically increased up to 44.6 fold, accompanied by reductions in EGCG, ECG, EGC, and EC in PFOTE (Figure 4B). The contents of major tea polyphynols in OTE, Pu-re tea extract (PTE), and PFOTE are compared in Table 2.

**Table 2:** Comparison of major tea polyphenol content in OTE, PTE, and PFOTE

mg/g	PFOTE	OTE	PTE-2
GA	237.7 ± 13.9	5.3 ± 0.1	115.0 ± 3.8
EGCG	14.3 ± 0.13	92.1 ± 0.6	2.8 ± 0.5
ECG	4.03 ± 1.0	20.2 ± 2.32	3.0 ± 0.1

### The beneficial effect of PFOTE on suppressing genomic DNA methylation and nuclear abundance of DNMTs in H1299 and A549 cells

**Figure 5:**
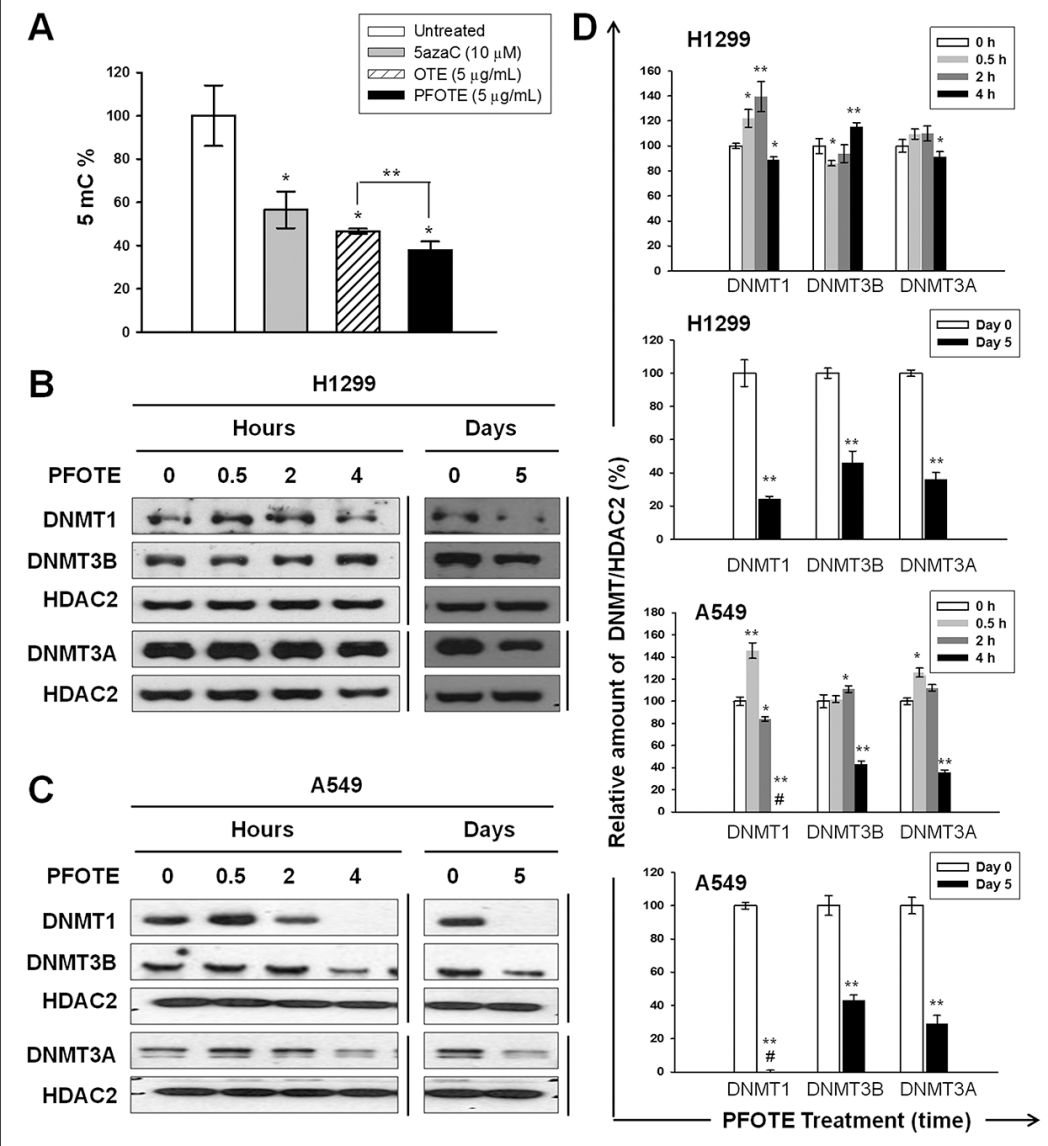
PFOTE changes the genomic 5mC content and nuclear accumulation of DNMTs in lung cancer cells lines (**A**) Cells were treated with OTE (5 μg/mL) or PFOTE (5 μg/mL) for 120 h; then genomic 5mC content analysis was conducted. 5azaC-treated cells were used as positive controls. (**B**–**C**) The amounts of nuclear DNMT1, DNMT3A, and DNMT3B were determined using western blotting at the indicated PFOTE (5 μg/mL) treatment times. (**D**) Normalization and quantification of the intensities of protein bands were performed as described in Figure 1.

Results from 5mC ELISA showed that PFOTE high in GA suppress DNA methylation to a greater extent than OTE (Figure 5A). The nuclear accumulation of DNMT1 increased at 0.5 and 2 h, followed by a drop at 4 h after 5μg/mL PFOTE treatment. The same treatment resulted in a slightly increased nuclear DNMT3B at 4 h post PFOTE treatment. The nuclear amount of DNMT3A remained almost unchanged. After cultivation in PFOTE for 5 d, we observed significant reductions in the nuclear accumulations of DNMT1, DNMT3A, and DNMT3B (Figure 5B, 5D). In the A549 cell line, both the short (4 h)- and long (5 days)-term treatments resulted in a complete absence of nucleus DNMT1, as well as in a significant reduction in nuclear DNMT3A and DNMT3B (Figure 5C, 5D). These data suggested that the fermentation process mediated by *A. sojae* may improve the epigenetic protective effects of OTE by inhibiting the abundance of nuclear DNMTs.

### PFOTE shows higher anti-cancer efficacy than OTE and enhances cisplatin-induced apoptosis

**Figure 6:**
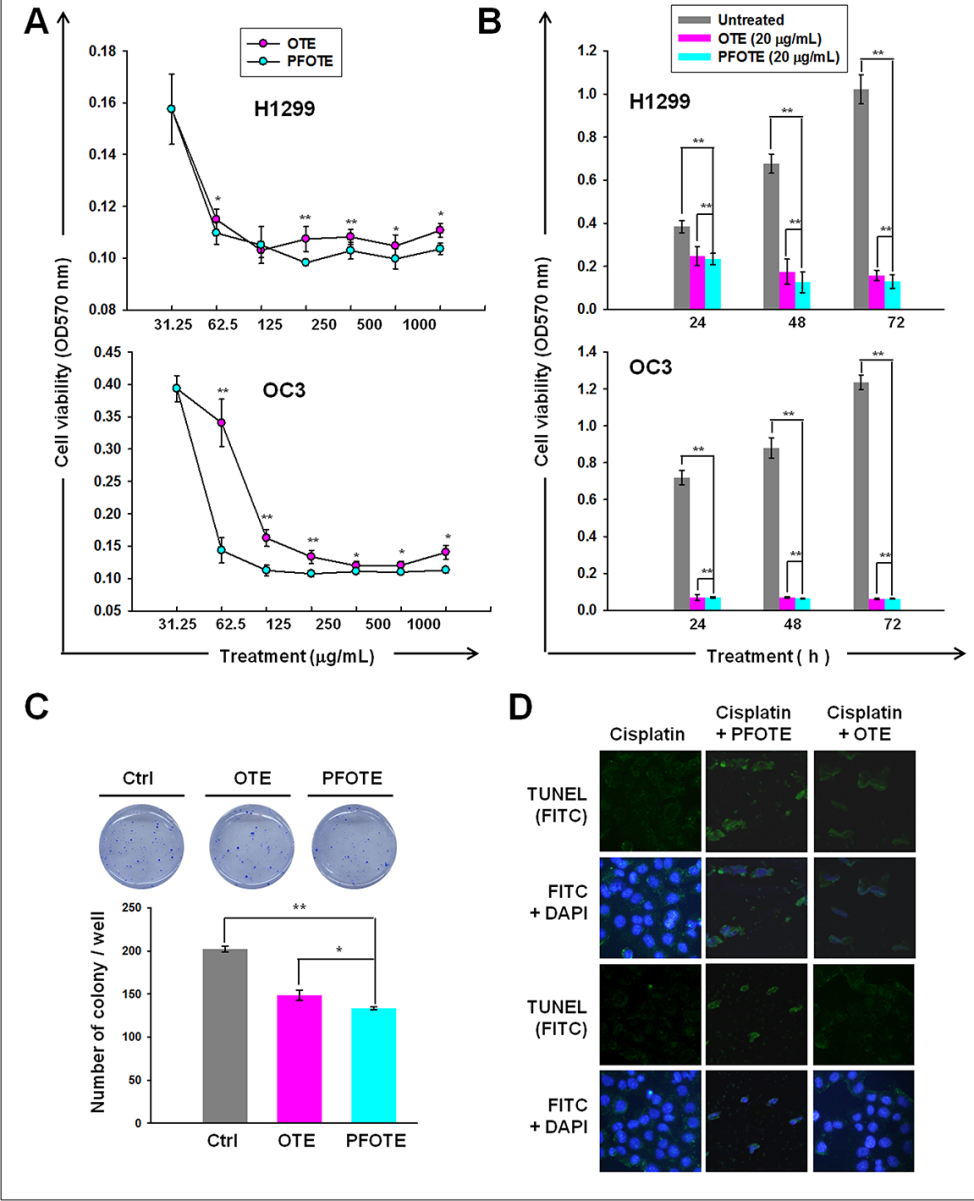
Anti-cancer effect of OTE and PFOTE on OC3 and H1299 cell lines (**A**–**B**) Cell viability was assessed by MTT after pre-treatment for 24 h with the indicated concentrations of tea extract (A) or treatment with 20 μg/mL tea extract at the indicated times (B). (**C**) The inhibitory effect of OTE and PFOTE on the anchorage-independent growth of H1299. (**D**) TUNEL assay was carried out in cisplatin-treated H1299 cells in the presence or absence of PFOTE or OTE for 24 h.

Treatment with either OTE or PFOTE resulted in the inhibition of cell proliferation in a dose- and time-dependent manner (Figure 6A, 6B). At 24 h, OTE and PFOTE suppressed H1299 cell viability with an IC_50_ of 388 μg/mL and 204 μg/mL, respectively; OTE and PFOTE suppressed OC3 cell viability with an IC_50_ of 235.4 μg/mL and 199.8 μg/mL, respectively. PFOTE exhibited a more potent cytotoxicity to H1299 than OTE at 48 h (*P* = 0.04), 72 h (*P* = 0.03), and 96 h (*P* < 0.000) post-treatment. For OC3 cells, PFOTE exhibited a significantly stronger cytotoxicity than OTE at all time points (*P* < 0.000) post-treatment (Figure 6B). Both OTE and PFOTE significantly (*P* < 0.001) reduced the numbers of anchorage-independent colonies in H1299 cells, and PFOTE had significantly (*P* = 0.04) greater inhibitory effects than OTE (Figure 6C). We performed an analysis of cisplatin-induced apoptosis using the TUNEL assay in the presence or absence of PFOTE or OTE. After drug exposure for 24 h, results revealed an apparent difference in sensitivity to cisplatin, with the number of TUNEL-positive cells obviously increased in H1299 cells co-treated with PFOTE, as compared to those co-treated with OTE or those without PFOTE or OTE treatment (Figure 6D).

## DISCUSSION

Given the reversibility of epigenetic modification, epigenetic therapy is a promising cancer treatment strategy to reprogram neoplastic cells toward an untransformed state. Several studies have suggested that phenolic compounds present in the human diet, which have long been used for medicinal purposes to prevent, relieve, or reverse carcinogenesis, are also promising agents for epigenetic therapy. DNMT1 is recognized as a key enzyme involved in the somatic inheritance of DNA methylation and thus plays an essential role in epigenome maintenance. In addition, Peters’ study has demonstrated a locus-specific function for DNMT1 in both maintenance and *de novo* promoter methylation [7]. In the present study, we demonstrate the inhibitory effect of GA on DNA methylation (Figure 1A). The nuclear DNMT1 almost disappeared after GA treatment for 7 days (Figure 1C). However, a decline in cytoplasmic DNMT1 also observed in GA-treated H1299, suggesting that GA reduces the protein stability of DNMT1. The nuclear translocation of DNMT1 has been found to be greatly enhanced by Akt-mediated phosphorylation [8]. In addition, activation of Akt may indirectly prolong the half-life of DNMT1 [3, 9]. DNMT1 protein stability is regulated via various post-translational modifications, such as methylation, phosphorylation, and ubiquitination, and also through protein–protein interactions [10]. DNMT1 binds with the histone methyltransferase Set7 and becomes methylated at K142. Such methylation triggers DNMT1 ubiquitination and subsequent degradation [9]. Phosphorylates DNMT1 at S143 by Akt unable Set7 to methylate DNMT1 at K142, in turn, lead to stabilizing DNMT1 [11]. As shown in Supplementary Figure 3, during 60–120 min of GA treatment phospho-Akt gradually decreased, accompanied by a reduction in phosphorylated DNMT1. This result suggests that GA exhibits a profound inhibitory effect on DNMT1 activity may through negatively regulating Akt phosphorylation, in turn reducing both the nuclear import and protein stability of DNMT1.

We also identified potential epigenetic targets of GA in H1299 cells (Table 1). Among them, four genes involved in the GADD45 signaling pathway (CCNE2, CCND3, CDKN1A, and CCNB1), were reactivated by promoter DNA methylation in the GA-treated lung cancer H1299 cells (Figure 3B–3E). This finding not only provides strong evidence for the influence of GA on DNA methylation but also increases the possibility that the anti-cancer capacity of dietary substances can be improved by GA enrichment because of the importance of GADD45 signaling in tumorigenesis [12]. In an attempt to develop a beverage for chemoprevention, by repeated screening and isolation processes, we identified *A. sojae*, which catalyzes the fermentation of tea and effectively produces GA from tea polyphenol, resulting in a specialized PFOTE high in GA. Compared with OTE, PFOTE exhibited more potent inhibitory effects on DNA methylation (Figure 5A). Moreover, PFOTE also showed greater cytotoxicity against lung and oral cancer cell lines (Figure 6B, 6C). These findings are particularly meaningful because of the specific relevance involving cigarette smoking and both lung and oral cancers and because of the severe epigenome alterations observed in cancer cells of the lung and oral tumors [1–3, 13, 14]. Furthermore, accumulating evidence has indicated the importance of epigenetic changes in carcinogenesis; virtually all cancer types have mutations in genes encoding many proteins regulating the epigenome [15–17]. Targeting the epigenome and including the use of inhibitors of DNA methylation and histone deacetylation [18–20] are evolving strategies for cancer chemoprevention, and both approaches have shown promise in cancer clinical trials. The ability of GA to target aberrant epigenomes (Figure 1), in addition to the anti-cancer mechanisms addressed by previous studies, makes it a valuable chemoprevention agent at multiple stages of malignant transformation.

Based on the degree of fermentation, tea can be classified into at least six types: green, yellow, white, oolong, black, and post-fermented tea [21]. Among them, post-fermented tea, such as pu’er, is unique because of the long-term microbial fermentation process, which may last from several months to many years [22]. Green and oolong teas have the highest content of EGCG, whereas pu’er tea has the highest content of GA [23]. The microbial fermentation process for producing pu’er is an integrated action of various molds, bacteria, and yeast; as a consequence, it is difficult to maintain consistency while preparing pu’er. In addition, there is great concern about fungal toxin contamination in pu’er. To overcome the disadvantages of pu’er, we developed a post-fermentation oolong tea high in GA by a 2-week fermentation process mediated by *A. sojae*, which preserves the anti-DNA methylation function of GA, showing stronger reduction effects on the genomic 5mC content in lung cancer cells than oolong tea and even the well-known DNMT inhibitor 5azaC (Figure 5A). Our data also showed that PFOTE treatment (5 μg/mL) for 5 days results in significant decreases in the cytoplasmic and nuclear abundance of all DNMTs in human lung cancer H1299 and A549 cell lines (Figure 5B), whereas a commercial pu’er tea extract exhibited almost negligible inhibitory effect on the nuclear abundance of DNMTs (Supplementary Figure 4), consistent with much less GA content in PTE (Table 2). These results clearly demonstrated the excellent effect of PFOTE in inhibition of DNMT activities. Based on the widespread prevalence of tea, post-fermentation oolong tea, proven by our work to be a dietary DNMT inhibitor, can be used as a daily beverage for cancer prevention by reducing the burden of epigenome aberrations. As shown in Figure 6D, combined treatment of PFOTE and cisplatin apparently enhanced the cisplatin-induced apoptosis in H1299 cells. These results suggested that, during the period of conventional medical treatment, drinking PFOT may increase the efficacy of anti-cancer chemotherapy or immune therapy without side effects.

To determine whether PFOTE is harmful to non-cancerous human cells, we examined the inhibition on viability by PFOTE with BEAS-2B, a cell line derived from the normal bronchial epithelium of non-cancerous individuals. As shown in Supplementary Figure 5, the results from MTT assay showed that the IC50s of PFOTE on H1299, OC3, and BEAS-2B cells were 204 ± 5.5, 199.8 ± 1.8, and 212.65 ± 3.2 mg/mL, respectively, after incubation for 24 h. Although the IC50 of PFOTE on BEAS-2B was slightly higher than those on the H1299 and OC3 cell lines, PFOTE exhibited a much more severe inhibitory effect on the viability of H1299 at a concentration of 31.25 mg/mL as compared to the effects on BEAS-2B and OC3 cells. Notably, at a concentration of 62.5 mg/mL, PFOTE appeared significantly less harmful to BEAS-2B than to both cancerous H1299 and OC3 cell lines. Because when post-fermentation oolong tea is used as a daily beverage its circulating levels is in the range of 5–10 μg/mL, we can expect that using post-fermentation oolong tea as a chemoprevention agent will not cause any adverse reactions to non-cancerous cells.

In summary, our findings not only reveal the potent epigenome protective function of GA but also provide a strong scientific foundation for the use of the specialized fermented oolong tea, generated by *A. sojae*-mediated biotransformation, as an effective dietary intervention strategy that is broadly applicable to public health recommendations involving tobacco-associated cancers.

## MATERIALS AND METHODS

### Materials

5-Azacytidine (5azaC), gallic acid (GA), (−)-gallocatechin (GC), (−)-epigallocatechin (EGC), catechin (C), caffeine (CAF), (−)-epigallocatechin gallate (EGCG), (−)-epicatechin (EC), (−)-gallocatechin-3-gallate (GCG), and (−)-epicatechin gallate (ECG) — were purchased from Sigma-Aldrich Co. USA. Anti-DNMT1 (GTX116011), anti- DNMT3A (GTX129125), and anti-DNMT3B (GTX129127) (GeneTex Co. Taiwan).

### Cell lines and cell culture

Human dysplastic oral keratinocyte DOK cell line [24] and human oral cancer cell line OC3 were maintained as previously described [1, 2]. Human non-small cell cancer cell lines A549 and H1299 (originally purchased from the ATCC) were maintained in DMEM and RPMI1640 medium, respectively, supplemented with 10% FBS, penicillin (100 U/mL) and streptomycin (100 μg/mL).

### Quantification of global DNA methylation

The global methylation of DNA was determined using a commercial kit (Cayman’s DNA Methylation EIA Kit) following the manufacturer’s protocol [1].

### Protein extraction and western blot analysis

Cells were lysed and centrifuged. Nuclear protein was harvested by using a Nuclear Extraction kit (Merck Millipore, MA, USA). Western blot analysis was performed as described previously [2].

### Gene expression microarray analysis

Extraction of total RNA, RNA quality evaluation, and cDNA microarray experiments were performed according to Affymetrix standard protocols by the microarray core laboratory at the National Health Research Institutes. The gene chips (Clariom S Assay HT06, Affymetrix Inc, CA, USA) were scanned with an Affymetrix Gene ChIP Scanner 3000 7G; and the CEL files generated were analyzed using Affymetrix Expression Console Software (version 1.4), which normalizes array signals using Signal Space Transformation (SST) and a robust multiarray averaging (RMA) algorithm. Normalized data were analyzed using Transcriptome Analysis Console (TAC) 3.0 software (Affymetrix). A paired *t*-test was applied to identify differentially expressed transcript genes between sample pairs and probes, with *P*-values less than 0.05 and fold-change ≥ 2 declared significant. Microarray expression data are available at the U.S. National Center for Biotechnology Information Gene Expression Omnibus (GEO) database under accession number GSE99993.

### Quantitative real-time qPCR (qPCR)

The synthesis of cDNA from total RNA and qPCR experiments were performed as described previously [2]

### Real-time quantitative methylation-specific PCR (qMSP)

Bisulfite conversions of genomic DNA and qMSP experiments were performed as described previously [2]. Primer sequences were provide in supporting information (Supplementary Table 1).

### Cell viability assay

Cells were seeded at 1 × 10^4^ per well in 96-well plates and incubated with different concentrations of EGCG or GA for 24 h, or with 5 μg/ml of OTE or PFOTE for the indicated time. Following incubation, MTT (Sigma-Aldrich) assay was performed as described previously [1]. The absorbance at 570 nm was measured on a micro-ELISA reader (Bio-Rad).

### BrdU cell proliferation assay

Cells were seeded in eight replicates in 96-well plates at a density of 1 × 10^4^ cells per well. After adhesion, cells were cultured in a standard culture medium or treated with indicated concentrations of GA, EGCG, or PFOTE for 48 h. Cell proliferation was evaluated by measuring BrdU incorporation during DNA synthesis according to the manufacturer’s instructions (Merck Millipore). BrdU incorporation was measured using chemiluminescence (absorbance: 450 nm).

### Soft agar assay

Soft agar assay was performed and quantified as described previously [2].

### Isolation and identification of fungus strain *Aspergillus sojae* (*A. sojae*)

Strain BCRC-30103 was isolated from oolong tea infusions. The purification and screening procedures were as described previously [25, 26]. Briefly, serial dilutions of oolong tea broth samples were plated to potato dextrose agar (PDA) and incubated at 30°C for 48 h–72 h. Single colonies with different shapes and colors were selected and transferred to potato dextrose broth (PDB), followed by incubation at 37°C for 24 h. After appropriate dilutions and re-plated to PDA, single colonies were picked for microscopic (Nikon E600, Japan) evaluation. Isolated strains were cultured on Tannin agar at 30 °C for 48 h for further analysis. Strain identification was accomplished by the Food Industry Research and Development Institute (FIRDI, Hsinchu, Taiwan).

### Preparation of oolong tea extract (OTE) and post-fermentation oolong tea extract (PFOTE)

A total of 1800 g of air-dried leaves were inoculated with *A. sojae* at 30°C for 2 weeks. The OTE and PFOTE were prepared as described previously [27–29].

### TdT-Mediated dUTP Nick End Labeling (TUNEL) Assay

Analysis of apoptosis was performed using TUNEL kit (Click-iT TUNEL Alexa Fluor Imaging Assay, Invitrogen) following the manufacturer’s protocol. Briefly, cells were plated at 2.5 × 10^5^ cells/six-well plate. After adhesion, the cells were treated with cisplatin (50 μM) alone or combined with either 5 μg/ml of PFOTE or OTE for 24 h before analysis. After fixation and permeabilization, cells were stained with TUNEL reaction mixture containing FITC-dUTP. Apoptotic nuclei (green) were observed under an Olympus inverted fluorescence microscope IX71S1F-3 (× 20).

### Statistical analysis

Comparison of the results between various experimentally treated groups and their corresponding controls was carried out by Student’s *t*-test analysis. When *p* < 0.05 (^*^) or *p* < 0.001 (^**^) differences between means were considered to be significant.

## SUPPLEMENTARY MATERIALS FIGURES AND TABLE



## Abbreviations

(GA): gallic acid
(PFOT): post-fermentation oolong tea
(OT): oolong tea
(PT): Pu’re tea
(PFOTE): post-fermentation oolong tea extract
(OTE): oolong tea extract
(PTE): Pu’re tea extract
(DNMTs): DNA methyltransferases
(DNMT1): DNA methyltransferases 1
(DNMT3A): DNA methyltransferases 3A
(DNMT3B): DNA methyltransferases 3B
