# Supplementary material for: The inhibitory activity of gallic acid against DNA methylation: application of gallic acid on epigenetic therapy of human cancers
Source: Oncotarget. 2017 Dec 7;9(1):361–74. doi: 10.18632/oncotarget.23015 (PMC5787471; doi:10.18632/oncotarget.23015)
Supplement: Supplementary file 1 [file oncotarget-09-361-s001.pdf]

The inhibitory activity of gallic acid against DNA methylation: application of gallic acid on epigenetic therapy of human cancers

SUPPLEMENTARY MATERIALS

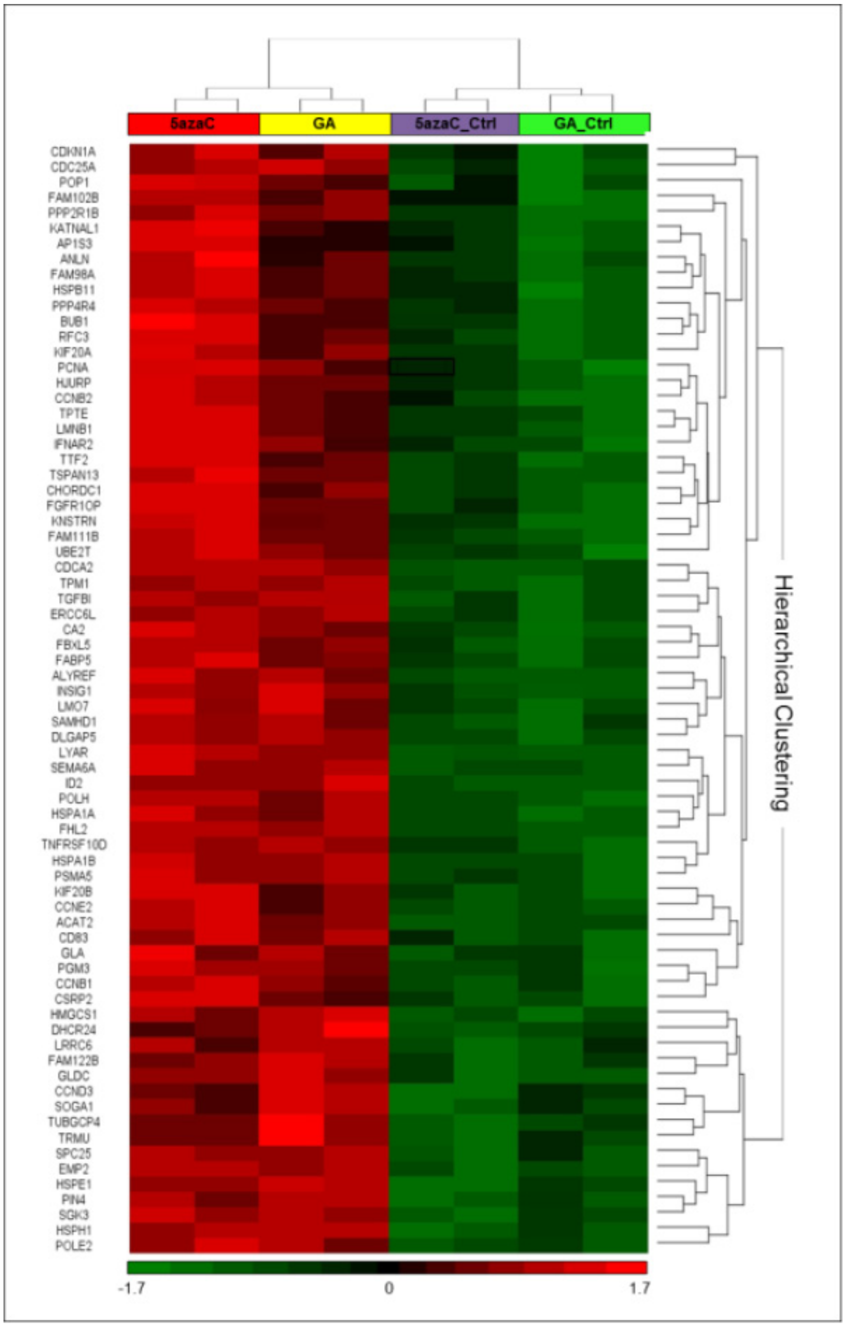

Supplementary Figure 1: Heat map showing relative expression of 72 genes that exhibited significant upregulated in gene expression following GA and 5azaC treatment. The heat map indicates up-regulation (red), down-regulation (green), and mean gene expression (black).

Rank	Score	Top function
1	44	Cell Cycle, DNA Replication, Recombination, and Repair, Cellular Compromise
2	34	Cellular Development, Cellular Growth and Proliferation, Renal Proliferation
3	22	Cellular Development, Cellular Growth and Proliferation, Cell Cycle
4	20	Cellular Development, Cellular Growth and Proliferation, Cell Death and Survival
5	2	Cancer, Cell Cycle, Cellular Development
6	2	DNA Replication, Recombination, and Repair, Nucleic Acid Metabolism, Small Molecule Biochemistry
7	2	Hereditary Disorder, Nephrosis, Organismal Injury and Abnormalities

Supplementary Figure 2: Ingenuity Pathway Analysis showing top biological functions of differentially expressed genes. .

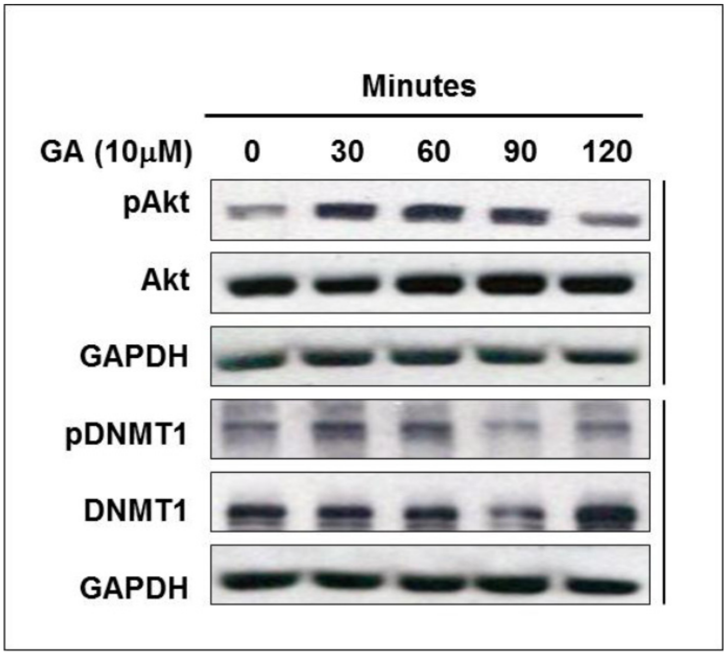

Supplementary Figure 3: A decline in phosphorylated Akt (pAkt) and phosphorylated DNMT1 (pDNMT1) after GA treatment. Cell lysates were harvested from H1299 cells treated with GA (10 μM) for the indicated time periods. The abundances of pDNMT1 and pAkt were determined using western blotting. The abundance of GAPDH detected on the same membrane (indicated by vertical lines) was used as a loading control for normalization.

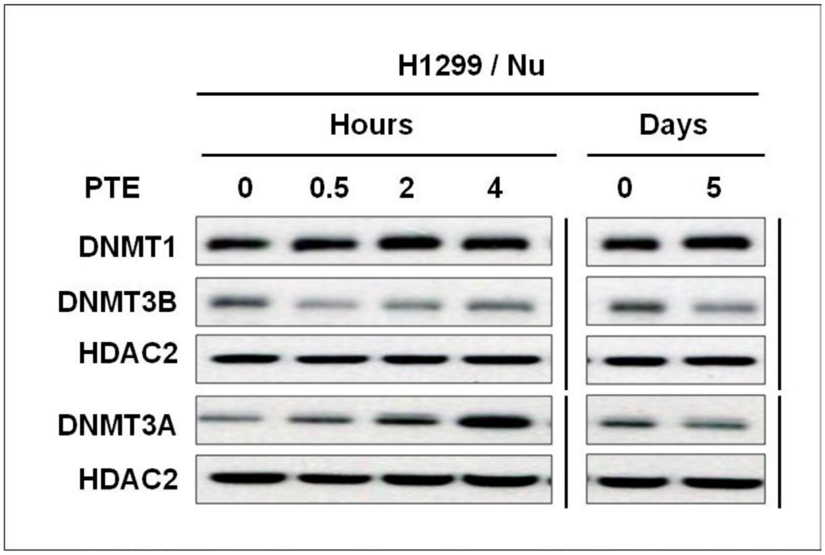

Supplementary Figure 4: Protein abundance of nuclear DNMTs in H1299 after Pu're tea extract (PTE) treatment for indicated time periods. The abundances of nuclear DNMT1, DNMT3A and DNMT3B were determined using western blotting at the indicated PTE (5 μg/mL) treatment times. The protein content of histone deacetylase 2 (HDAC2) detected on the same membrane (indicated by vertical lines) was used as an internal control for DNMT1, DNMT3B, and DNMT3A

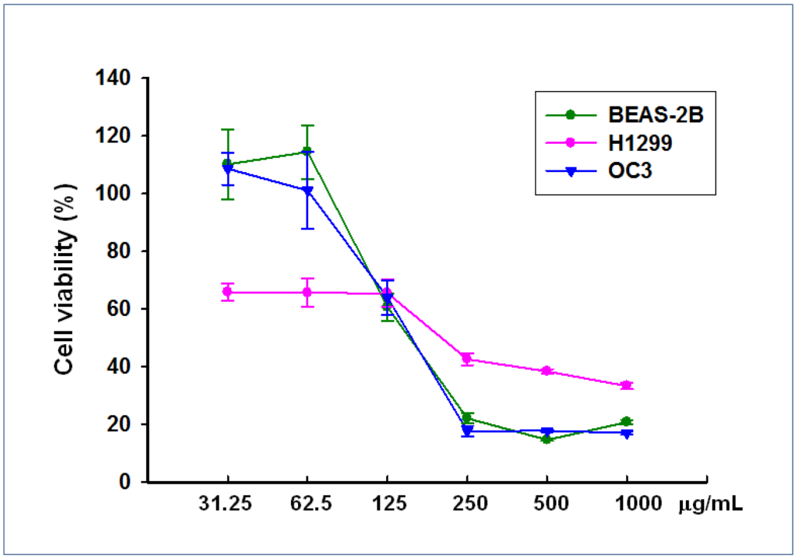

Supplementary Figure 5: Inhibitory effect of PFOTE on viability of BEAS-2B, H1299, and OC3 cell lines. Cell viability was assessed by MTT method after 24-h pre-treatment with the indicated concentrations of PFOTE.

Supplementary Table 1: Primer sequences for qMSP (A) and qPCR (B)

(A)

Primers for qMSP		
**Gene symbol**		**Sequences**
CCNE2	F	5′ GTCGTATTTGGTTAGGCGC 3′
	R	5′ TTTCCTAAACGACGCGAA 3′
CCND3	F	5′ TATTTTGAGAGGTCGAGGC 3′
	R	5′ CTAAAACTACAAACGCACGC 3′
CDKN1A	F	5′ GAGTCGAGTTAAGCGTGTTC 3′
	R	5′ AAAAACACCGTATACGCAAA 3′
CCNB1	F	5′ GTTTCGGATTGCGAATTAAC 3′
	R	5′ CACCTCGACGCTCTCTTAAT 3′

(B)

Primers for qPCR		
**Gene symbol**		**Sequences**
CCNE2	F	5′ GCCATTGATTCATTAGAGTTCCA 3′
	R	5′ CTGTCCCACTCCAAACCTG 3′
CCND3	F	5′ GGTCACCTGACGAGGAGGTA 3′
	R	5′ GGTAGCGATCCAGGTAGTTCA 3′
CDKN1A	F	5′ CCGAAGTCAGTTCCTTGTGG 3′
	R	5′ CATGGGTTCTGACGGACAT 3′
CCNB1	F	5′ CATGGTGCACTTTCCTCCTT 3′
	R	5′ AGGTAATGTTGTAGAGTTGGTGTCC 3′
